# IRF4 affects the protective effect of regulatory T cells on the pulmonary vasculature of a bronchopulmonary dysplasia mouse model by regulating FOXP3

**DOI:** 10.1186/s10020-023-00770-y

**Published:** 2024-01-09

**Authors:** Ying Zhu, Langyue He, Yue Zhu, Huici Yao, Jianfeng Jiang, Hongyan Lu

**Affiliations:** Department of Pediatrics, Affiliated Hospital of Jiangsu University, Zhenjiang, China

**Keywords:** Bronchopulmonary dysplasia, Pulmonary vascular development, Pulmonary vascular endothelial cells, Regulatory T cells, Interferon regulatory factor 4, Forkhead box protein P3

## Abstract

**Background:**

Bronchopulmonary dysplasia (BPD) is a common chronic lung disease in preterm infants, characterised by compromised alveolar development and pulmonary vascular abnormalities. Emerging evidence suggests that regulatory T cells (Tregs) may confer protective effects on the vasculature. Knockdown of their transcription factor, interferon regulatory factor 4 (IRF4), has been shown to promote vascular endothelial hyperplasia. However, the involvement of Tregs and IRF4 in the BPD pathogenesis remains unclear. This study aimed to investigate the regulation of Tregs by IRF4 and elucidate its potential role in pulmonary vasculature development in a BPD mouse model.

**Methods:**

The BPD model was established using 85% hyperoxia exposure, with air exposure as the normal control. Lung tissues were collected after 7 or 14 days of air or hyperoxia exposure, respectively. Haematoxylin–eosin staining was performed to assess lung tissue pathology. Immunohistochemistry was used to measure platelet endothelial cell adhesion molecule-1 (PECAM-1) level, flow cytometry to quantify Treg numbers, and Western blot to assess vascular endothelial growth factor (VEGFA), angiopoietin-1 (Ang-1), forkhead box protein P3 (FOXP3), and IRF4 protein levels. We also examined the co-expression of IRF4 and FOXP3 proteins using immunoprecipitation and immunofluorescence double staining. Furthermore, we employed CRISPR/Cas9 technology to knock down the *IRF4* gene and observed changes in the aforementioned indicators to validate its effect on pulmonary vasculature development in mice.

**Results:**

Elevated IRF4 levels in BPD model mice led to FOXP3 downregulation, reduced Treg numbers, and impaired pulmonary vascular development. Knockdown of *IRF4* resulted in improved pulmonary vascular development and upregulated FOXP3 level.

**Conclusion:**

IRF4 may affect the protective role of Tregs in the proliferation of pulmonary vascular endothelial cells and pulmonary vascular development in BPD model mice by inhibiting the FOXP3 level.

## Background

Bronchopulmonary dysplasia (BPD) is a chronic lung disease characterised by impaired alveolar and pulmonary vascular development. Its pathogenesis remains poorly understood and primarily affects preterm infants exposed to various prenatal or postnatal factors, including intrauterine infection, mechanical ventilation, oxygen therapy, and inflammation (Doyle et al. 2021; Gilfillan et al. 2021). Hyperoxia exposure is a significant contributor to BPD, activating pro-inflammatory factors and inflammatory mediators via oxidative stress. This leads to impaired pulmonary vascular formation and branching, resulting in pulmonary vascular injury and dysplasia, often leading to pulmonary hypertension (Jobe et al. 2001; Wang et al. 2020; Gentle et al. 2023). Severe BPD can be associated with pulmonary hypertension and associated cardiovascular diseases, leading to lifelong respiratory disease. Pulmonary vascular endothelial cells (PVECs) are an important component of pulmonary vasculature growth and development through proliferation, migration, and tube formation. They are a primary target of oxidative stress (Zhang et al. 2019). Platelet endothelial cell adhesion molecule-1 (PECAM-1) serves as a proliferation marker for PVECs, while vascular endothelial growth factor (VEGFA) and angiopoietin-1 (Ang-1) regulate PVEC function and promote pulmonary vascular development (You et al. 2020; Perrone et al. 2023; Sun et al. 2013).

Regulatory T cells (Tregs), a subset of immunosuppressive T cells, have shown protective effects on vasculature in conditions such as pulmonary arterial hypertension. However, the impact of Tregs on pulmonary vascular development in BPD, particularly due to hyperoxia exposure, and the underlying mechanisms remain unclear (Tamosiuniene et al. 2018). Forkhead box protein P3 (FOXP3), a core transcription factor of Tregs, plays a pivotal role in regulating Treg development and function (Ohkura et al. 2020). Xu et al. demonstrated that decreased FOXP3 level is associated with reduced tumour angiogenesis (Xu et al. 2020). Impaired Treg function due to reduced FOXP3 level may contribute to pulmonary vascular remodelling and subsequent pulmonary hypertension (Chen et al. 2023). Beyond FOXP3, various co-transcription factors also regulate Treg differentiation. Additionally, FOXP3 has been found to promote pulmonary angiogenesis and development in BPD model mice and is negatively correlated with the protein level of the interferon regulatory factor 4 (IRF4), a co-transcription factor of Treg. IRF4 is broadly expressed in immune system cells and has been linked to conditions such as asthma, inflammatory bowel disease, and autoimmune diseases (Shi et al. 2020; Xu et al. 2012). Sequencing analyses have identified IRF4 as an important Treg transcription factor primarily involved in immunomodulation. Some studies have indicated that IRF4 might regulate Treg expression by acting on the *FOXP3* gene (Hwang et al. 2010; Trujillo-Ochoa et al. 2023; Dominguez-Villar et al. 2018). For instance, Wenlin et al. indicated that IRF4 may be involved in the regulation of vascular intimal hyperplasia by acting on smooth muscle cells and macrophages, and the *IRF4*^−/−^ mouse model can significantly promote intimal hyperplasia and contribute to angiogenesis (Cheng et al. 2017).

Nonetheless, whether Tregs offer protective effects on pulmonary vascular development in BPD model mice and whether this effect is related to immune regulation between IRF4 and FOXP3 remains unclear. In this study, we investigated the effects of Tregs and the mechanism of action of its transcription factors IRF4 and FOXP3, on both the proliferation of PVECs and pulmonary vascular development by establishing a hyperoxia-exposure mouse BPD model.

## Materials and methods

### Experimental animals

All experimental animal protocols were reviewed by the the Experimental Animal Management and Use Committee of Jiangsu University.Twenty-four C57BL/6 pregnant mice (gestation 17–18 d) were procured from the Experimental Animal Centre of Jiangsu University. In order to exclude the influence of sex differences on the results of the study, we chose male neonatal mice for the study. Newborn mice delivered from C57BL/6 pregnant mice, six male neonatal mice were randomly selected from each group and divided into normoxia for 7 d, hyperoxia for 7 d, normoxia for 14 d, and hyperoxia for 14 d. *IRF4*^−/−^ mice were obtained from Jiangsu Jicui Pharmachem Bio-technology. Two female *IRF4*^*−/−*^ mice and six male *IRF4*^*−/−*^ mice were caged together, and after pregnancy was confirmed, the *IRF4*^*−/−*^ pregnant mice (17–18 d of gestation) were housed individually, and six male neonatal mice were randomly selected as the *IRF4*^*−/−*^ group.

### BPD model construction and grouping

Using hyperoxia exposure to simulate the BPD model, mice were randomly divided into an air control group and a hyperoxia group. The hyperoxia group was placed in an oxygen tank with 85% oxygen concentration, while the control group was exposed to atmospheric air in the same room. To prevent oxygen toxicity and eliminate the influence of surrogate lactating mothers, surrogate mothers were swopped between the air and hyperoxia groups every 12 h. The conditions of the mice and mothers were monitored and recorded daily (Yao et al. 2023). At 7 d and 14 d after respective exposures, six mice in each group were anaesthetised, tracheal intubated, fixed in situ with 4% paraformaldehyde intratracheal injection for 10 min, and the lung lobe tissue was removed. The lung lobe tissue was stored in the refrigerator at − 80 °C and used for related experimental studies.

### Histopathological examination of the lungs

Paraffin-embedded lung tissues were serially sectioned at a thickness of 4 μm, stained with HE, and the morphological changes were observed under the microscope.

### Immunohistochemistry (IHC) detection of PECAM-1 expression in lung tissue

Lung lobe tissue sections at each time point for each group were routinely processed for antigen retrieval, endogenous peroxidase blocking, bovine serum albumin(BSA) closing, and incubated with rabbit-derived primary antibody anti-PECAM-1 (1:300; Abcam, Cambridge, UK), followed by secondary anti-IgG (1:200; Abcam, Cambridge, UK). Staining was performed with DAB, counterstained with haematoxylin, and dehydrated to seal the sections. Three non-overlapping areas were independently selected by two senior pathologists in each group, totalling six non-overlapping areas. Analysis was performed using the Image J acquisition system: brownish-yellow particles in the cells were taken as positive staining, and the positive cell integral optical density value was recorded as the PECAM-1 content of that lung tissue specimen. The pulmonary vascular density was calculated as follows: pulmonary vascular density (%) = the area occupied by the endothelial cells of the lung tissue immunostained positively for PECAM-1/the total area of the lung parenchyma cells × 100% (Hussnain and Shi 2021).

### Western blot detection of VEGFA, Ang-1, FOXP3, and IRF4 protein level in lung tissue

RIPA lysate (Beijing Kangwei Century Company Biotechnology, Beijing, China), containing PMSF and protease inhibitors (Shanghai Biyuntian Biotechnology, Shanghai, China) was used to extract the total proteins of the 20 mg lung lobe tissue cells of each group. Total protein concentration of about 3 μg/μL by BCA method and 10 μL total proteins were taken from each lane and subjected to sodium dodecyl sulfate–polyacrylamide gel electrophoresis(SDS-PAGE). Subsequently, the proteins were wet-transferred to a polyvinylidene fluoride membrane and blocked at 37 °C for 1 h with 5% skimmed milk powder. Primary antibodies, including anti-VEGFA (1:500; Abcam, Cambridge, UK), anti-Ang-1 (1:2,000; Abcam, Cambridge, UK), anti-FOXP3 (1:500; Santa Cruz Biotechnology, Santa Cruz, CA, USA), anti-IRF4 (1:1,000; Cell Signaling Technology, Danvers, MA, USA), and β-actin (1:1,000; Cell Signaling Technology, Danvers, MA, USA) were added respectively, and incubated at 4 °C overnight. After washing the membrane, secondary antibody IgG (1:5000; Immunoway, Plano, Texas, USA) was added, and the membrane was incubated at 37 °C for 1 h. Enhanced chemiluminescence was used for visualisation. The relative level of the target protein was determined as the ratio of the grey value of the target protein to the β-actin band.

### Co-immunoprecipitation (Co-IP) to detect IRF4 and FOXP3 co-expression in lung tissue

Mouse lung lobe tissue weighing 0.1 g was homogenised in 1 mL Non-Denaturing Lysis Buffer (Abbkine, Wuhan, China, Wuhan, China) with 1 μL Proteinase Inhibitor Cocktail (Abbkine, Wuhan, China) on ice for 5 min. Following centrifugation at 12,000 rpm for 10 min at 4 ℃, and the total protein concentration was determined to be about 3 μg/μL by BCA method. Then, 20 μL supernatant was taken as input and added to 5 × SDS-PAGE Loading Buffer and boiled for 10 min. A total of 20 μL Protein A/G Magnetic Beads (Abbkine, Wuhan, China) was added to each centrifuge tube and placed on the magnetic separation rack. The supernatant was aspirated and discarded, and 1 mL 1 × Wash Buffer (Abbkine, Wuhan, China) was added to the tube. After resuspending and washing the beads three times with 1 × Wash Buffer (Abbkine, Wuhan, China), 10 μL FOXP3 antibody and 2 μL Mouse IgG (Abbkine, Wuhan, China) were added respectively. The mixture was incubated for 30 min at 25 °C with shaking. Following washing and discarding the supernatant three times, 0.25 mL protein supernatant was added and incubated overnight at 4 °C. Then, 30 μL 1 × SDS-PAGE Loading Buffer was added, and the mixture was heated at 100 °C for 5 min. After centrifugation at 800 rpm for 1 min, the supernatant was collected for Western Blotting.

### Immunofluorescence double staining detection of IRF4 and FOXP3 co-localisation

After fixing mouse lung tissue with 4% paraformaldehyde, paraffin-embedded, serial slices were cut at a thickness of 4 μm, and the slices were baked at 60 ℃ for 1 h. The slices were deparaffinized by means of xylene and gradient alcohols, and antigenic repair was performed by the high-pressure method. Then the endogenous peroxidase activity was blocked by incubation with 3% H2O2 at room temperature for 10 min.The sections were incubated with 5% BSA for 20 min for blocking and then washed 3 times with phosphate buffer solution (PBS) for 5 min/session. Next, they were incubated with two antibodies, anti-FOXP3 (1:200;Santa Cruz Biotechnology, Santa Cruz, CA, USA) and anti-IRF4 (1:100;Cell Signaling Technology, Danvers, MA, USA), overnight at 4 °C. The sections were allowed to warm up at 37 °C for 30 min the following day, and then incubated with secondary antibodies mouse IgG and rabbit IgG (1:1,000; Abcam, Cambridge, UK) for 1 h. After washing with PBS three times, the sections were stained with DAPI (Abbkine, Wuhan, China) for nuclei visualisation. Immunofluorescence staining was observed using a fluorescence microscope. Red fluorescence indicated positive expression of FOXP3, green fluorescence indicated positive expression of IRF4, and blue fluorescence represented DAPI-labelled nuclei. Co-localisation of FOXP3 and IRF4 was depicted in orange.

### Flow cytometry for measuring Treg count as well as IRF4 and FOXP3 transcription factor expression in lung tissue

Mouse lung lobe tissue weighing 40 mgwas taken from each group at each time point. The tissue was rinsed with PBS, mechanically sheared, ground, and then passed through a 70 μm cell filter. After erythrocytes lysis using ammonium-chloride-potassium lysis buffer (Leagene, Beijing, China, Beijing, China), the cell suspension from a single mouse was used for T-lymphocyte isolation with a mouse organ tissue lymphocyte isolation solution kit (Solarbio, Beijing, China). The isolated cells were washed with PBS, centrifuged, and stained with surface antibodies using PE anti-cluster of differentiation 4(CD4)(Thermo Fisher Scientific, Waltham, MA, USA), APC anti-cluster of differentiation 25(CD25) (Thermo Fisher Scientific), and PE-cy7 anti-IRF4 (Cell Signaling Technology, Danvers, MA, USA) for 45 min. After that, the cells were fixed and permeabilised with a cell-fixing and permeabilisation reagent for 1 h. Subsequently, intranuclear cytokine AF488 anti-FOXP3 (Thermo Fisher Scientific, Waltham, MA, USA) staining was performed. Cells labelled as CD4^+^ CD25^+^ FOXP3^+^ were identified as Tregs, and their expression was assessed using flow cytometry (BD FACS Canto; BD, Franklin Lake, NJ, USA). Flow Jo v10 (BD) software was used for data analysis.

### Construction and grouping of ***IRF4***^−/−^ mice

The CRISPR/Cas9 technology was used to knock down the *IRF4* gene. Exons 3–5 of the Irf4-201 transcript in the *IRF4* gene structure were selected as the knockdown region, which contains a 421 bp coding sequence. Knockdown of this region results in disruption of protein function. Based on available MGI data, purebred mice with disruption of this gene exhibited abnormalities in the immune system, including T and B cell development, and have altered susceptibility to bacterial and viral infections. They also demonstrate impaired thermogenic gene expression and energy expenditure.

*IRF4*^−/−^ male and female mice were mated in a 3:1 ratio within combined cages. After confirming pregnancy, mice delivered from *IRF4*^−/−^ pregnant mice (16–17 d of gestation) and C57BL/6 pregnant mice (16–17 d of gestation) were divided into *IRF4*^−/−^ and C57 groups. Both groups were placed in an oxygen chamber with the oxygen concentration maintained at 85%, and surrogate lactating females were changed every 12 h. The mice were exposed to high oxygen for 14 d following exposure. At 14 d after exposure to hyperoxia, lung tissues from six mice in each group were used for subsequent experimental studies.

### Statistical analyses

Data were subjected to statistical analysis using GraphPad Prism v8.0 (GraphPad Software, San Diego, CA, USA). Measurement information was expressed as mean ± standard deviation(SD), and a *t*-test was used for comparison between the two samples. The correlation between the two samples was analysed by Pearson correlation analysis. A *p*-value less than 0.05 was considered statistically significant.

## Results

### Impaired pulmonary vascular development in BPD model mice, along with decreased Treg and FOXP3 expression and increased IRF4 expression

**Fig. 1:**
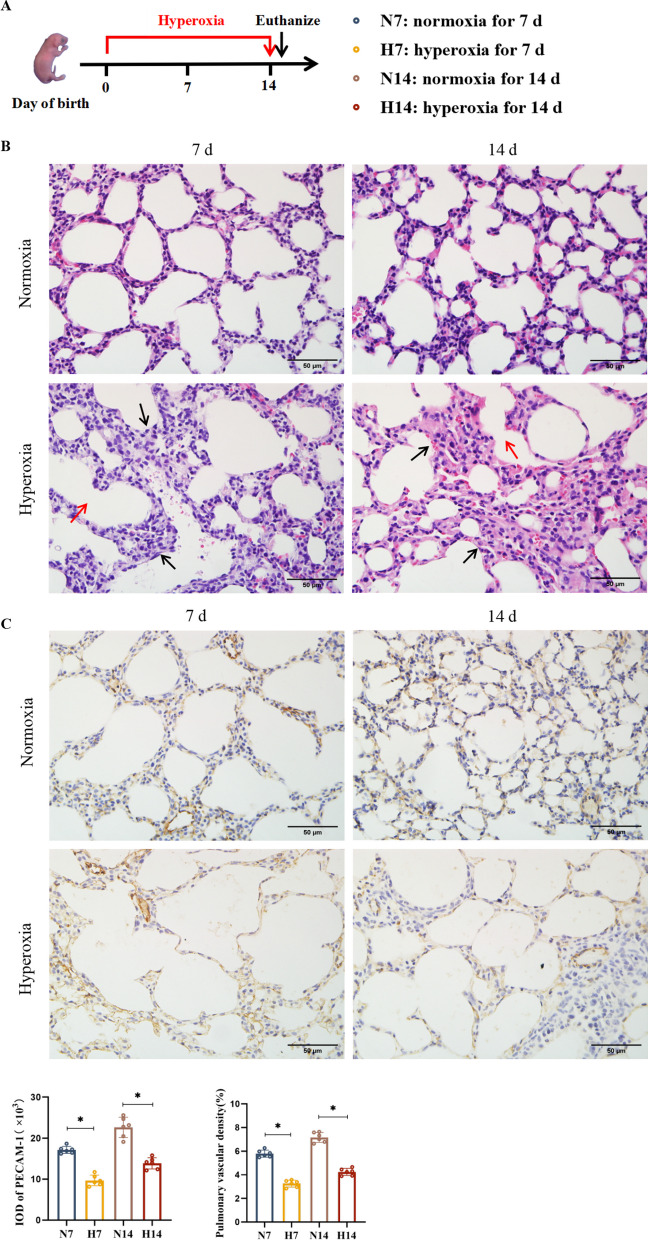

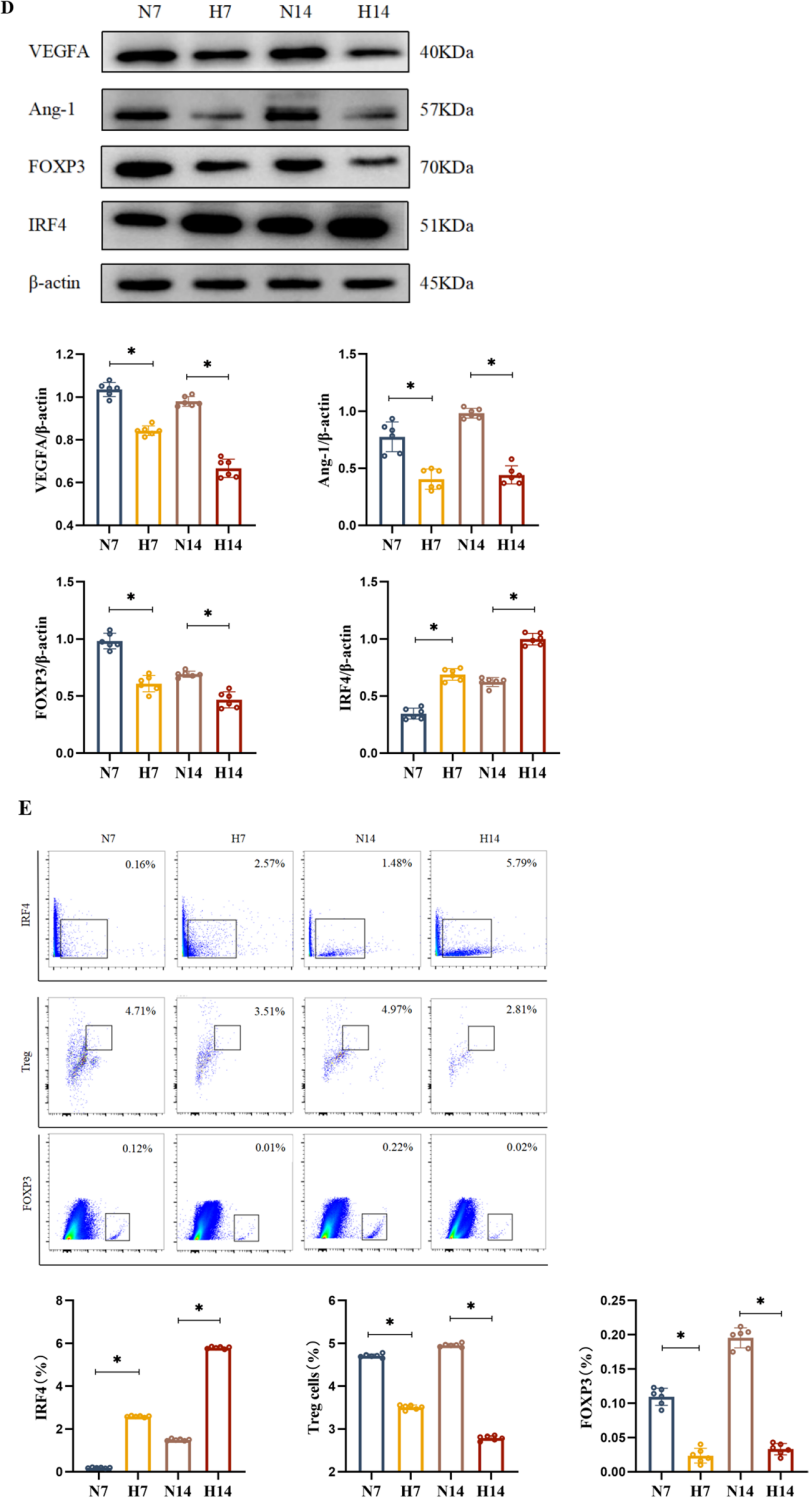

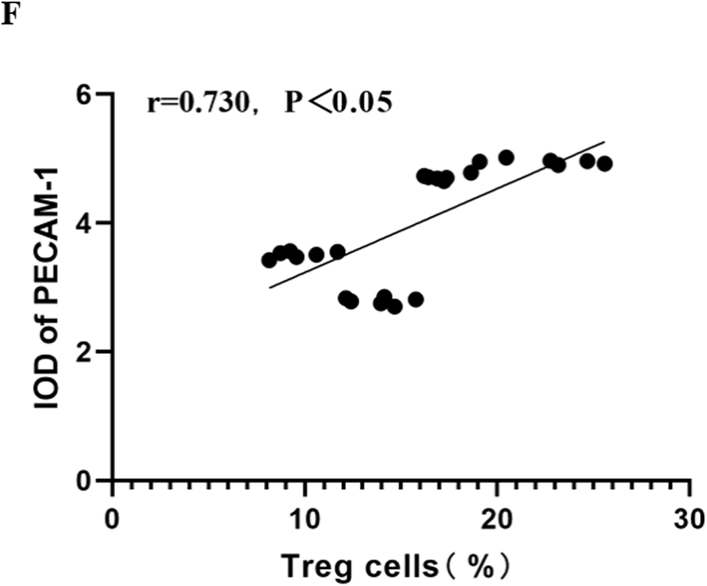
Impaired pulmonary vascular development in BPD model mice, with increased IRF4 and decreased FOXP3 protein levels and number of Tregs. **A** BPD model simulated using hyperoxia exposure, and mouse lung tissues taken at 7 d and 14 d after air or hyperoxia exposure, respectively. **B** HE staining to observe the morphology of mouse lung tissues (× 400; scale bar, 50 μm). Black arrows refer to thickened alveolar septa, red arrows refer to fused and enlarged alveolar lumina. **C** IHC of PECAM-1 level in mouse lung tissues (× 400; scale bar, 50 μm). **D** Western blot of VEGFA, Ang-1, FOXP3, and IRF4 protein levels in mouse lung tissue. **E** Flow cytometry to detect the number of Tregs and the expression of IRF4 and FOXP3 in the lung tissues of the mice. **F** Pearson correlation analysis to derive a positive correlation between the number of Tregs and the level of PECAM-1 (r = 0.730). Data are expressed as mean ± SD. n = 6. Significant differences are indicated by *p < 0.05

The BPD model was established by hyperoxia exposure (Fig. 1A). HE staining results showed that lung tissues from the hyperoxia group, compared to the control group at corresponding time points, displayed structural disorganisation, thickened lung septa, enlarged and fused alveoli with a reduced number and varying sizes. These findings were consistent with classical BPD pathological characteristics, confirming the success of the model (Fig. 1B). IHC results indicated a decrease in PECAM-1-positive cell integral optical density and pulmonary vascular density in the hyperoxia group (Fig. 1C). Furthermore, Western blot results showed significantly reduced protein levels of VEGFA and Ang-1 in the lung tissues of hyperoxia-exposed mice at 7 d and 14 d postnatally compared with the control group (Fig. 1D).

To investigate whether the Treg counts changed in the BPD model mice, we used flow cytometry. The results showed a decrease in Treg numbers at all time points in the hyperoxia group compared to the control group at the corresponding points (Fig. 1E). Moreover, changes in Treg number were consistent with trends observed in the PECAM-1 mean integral optical density values and the protein levels of VEGFA and Ang-1. To further clarify whether Tregs were related to the proliferation of PVECs, correlation analysis further indicated a positive relationship between Treg numbers and PECAM-1 expression (Fig. 1F).

To determine whether the level of FOXP3 correlated with Treg numbers, flow cytometry was used to detect FOXP3 counts. The results indicated a decrease in FOXP3 factors at all time points in the hyperoxia group compared to the control group at the same time points (Fig. 1E). Western blot results also showed reduced FOXP3 protein levels at all time points in the hyperoxia group compared to the control group, aligning with the trend in Treg levels (Fig. 1D).

We used both flow cytometry and Western blot to detect IRF4 quantity and protein level. Flow cytometry results demonstrated an increase in IRF4 counts at all time points in the hyperoxia group compared with the control group at the corresponding time points (Fig. 1E). Similarly, Western blot results indicated significantly higher IRF4 protein levels in the lung tissues of the hyperoxia group compared to the control group at the same time points (Fig. 1D), confirming upregulation of IRF4 in the BPD model mice.

### IRF4 inhibited FOXP3 level

**Fig. 2:**
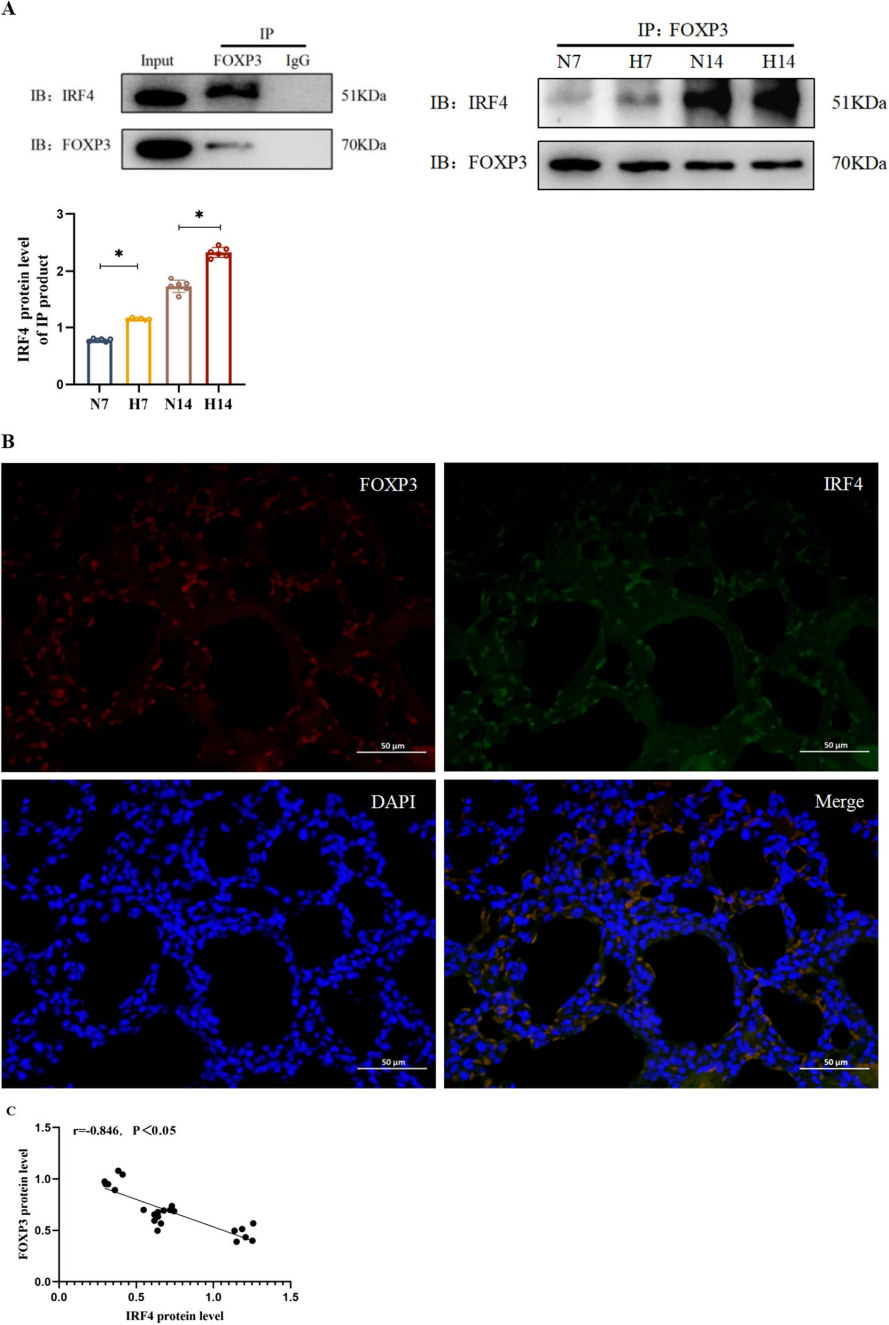
IRF4 inhibited the expression of FOXP3. **A** Co-IP detection of IRF4 and FOXP3 co-expression in lung tissues. **B** Immunofluorescence double staining detection of IRF4 and FOXP3 co-localisation (× 400; scale bar, 50 μm). Red fluorescence indicated positive expression of FOXP3, green fluorescence indicated positive expression of IRF4, and blue fluorescence represented DAPI-labelled nuclei. Co-localisation of FOXP3 and IRF4 was depicted in orange. **C** Pearson correlation analysis to show a negative correlation between IRF4 and FOXP3 protein level (r = − 0.846). Data are expressed as mean ± SD. n = 6. Significant differences are indicated by *p < 0.05

To investigate whether there were interactions between IRF4 and FOXP3, we conducted experiments on mouse lung tissues exposed to hyperoxia for 14 d. Immunoprecipitation results confirmed protein interactions between IRF4 and FOXP3 (Fig. 2A). Additionally, immunofluorescence results also showed partial co-localisation of FOXP3 and IRF4 (Fig. 2B). Flow cytometry and Western blot results indicated that IRF4 protein level increased, while FOXP3 level decreased in the hyperoxia group. Correlation analysis further demonstrated a negative correlation between the IRF4 protein level and the FOXP3 level (Fig. 2C), suggesting that IRF4 inhibited the FOXP3 protein level.

### Knockdown of *IRF4* increased FOXP3 expression and Treg numbers and reduced hyperoxia-induced impaired pulmonary vascular development and lung injury

**Fig. 3:**
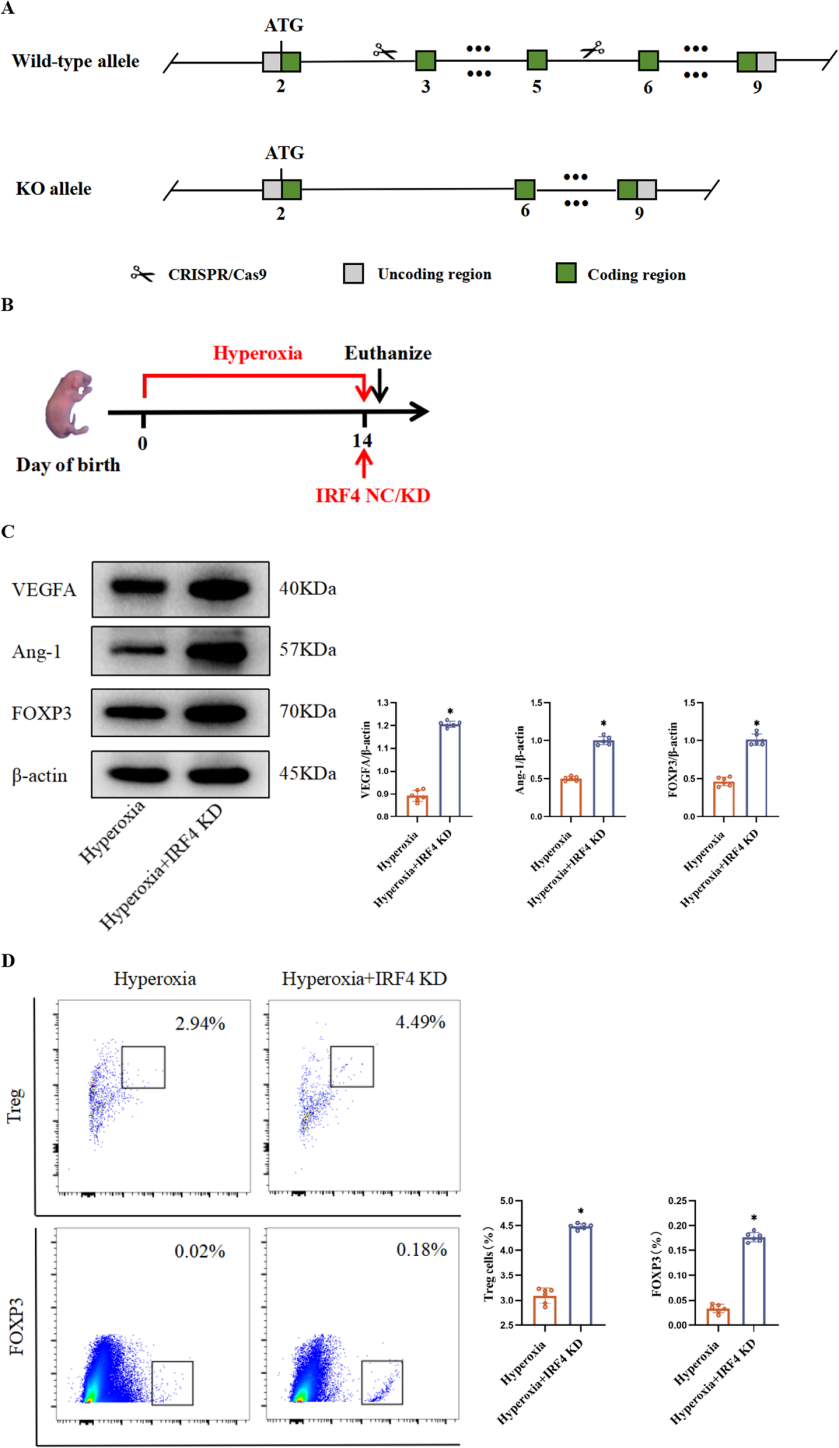

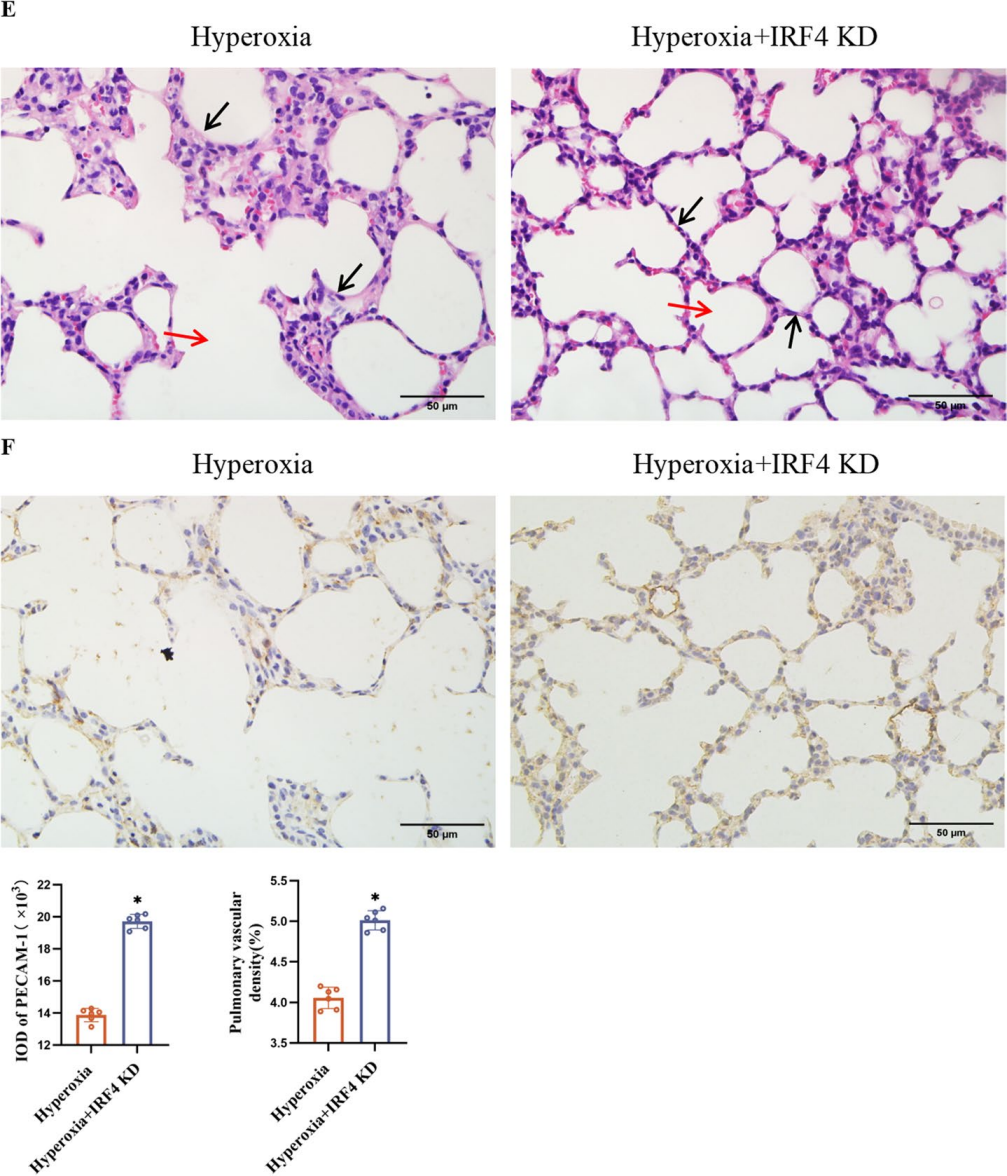
*IRF4*^−/−^ mice with increased FOXP3 level and Treg numbers show reduced pulmonary vascular dysplasia and lung injury. **A** Demonstration of gene ablation.In this project we use CRISPR/Cas9 technology to modify *IRF4* gene. The *IRF4* gene has 3 transcripts. According to the structure of *IRF4* gene, exon3-ex on5 of *IRF4*-201(ENSMUST00000021784.9) transcript is recommended as the knockout region.The region contains 421 bp coding sequence. Knock out the region will result in disruption of protein function. **B** C57 and *IRF4*.^−/−^ mice exposed to hyperoxia for 14 d. Mouse lung tissues are used for experiments. **C** Western blot of VEGFA, Ang-1 and FOXP3 protein levels in mouse lung tissues. **D** Flow cytometry of the number of Tregs and FOXP3 level in mouse lung tissues. **E** HE staining of the morphology of mouse lung tissues (× 400; scale bar, 50 μm). Black arrows refer to alveolar septa, red arrows refer to alveolar lumina. **F** IHC of PECAM-1 level in mouse lung tissue (× 400; scale bar, 50 μm). Data are expressed as mean ± SD. n = 6. Significant differences are expressed as *p < 0.05

To determine whether IRF4 affected FOXP3 level and Treg proliferation in BPD model mice, we employed CRISPR/Cas9 technology to knock down the *IRF4* gene (Fig. 3A). Both C57 mice and *IRF4*^−/−^ mice were exposed to hyperoxia for 14 d (Fig. 3B). Western blot results demonstrated a significant increase in FOXP3 protein level in the lung tissues of *IRF4*^−/−^ mice compared with C57 mice (Fig. 3C). Furthermore, flow cytometry results showed an increased Treg count in the lung tissues of *IRF4*^*−/−*^ mice compared with C57 mice (Fig. 3D).

To study the effect of *IRF4* knockdown on pulmonary vascularity and lung development in hyperoxia-induced mice, we conducted HE staining on lung tissues from *IRF4*^*−/−*^ mice exposed to hyperoxia for 14 d. Results showed that *IRF4*^−/−^ mice exhibited smaller alveolar lumens, increased alveolar number, thinner septa, and improved lung histopathology compared to C57 mice at the same time point (Fig. 3E). Additionally, IHC results showed elevated PECAM-1-positive cell integral optical density values and pulmonary vascular density in the lung tissues of *IRF4*^−/−^ mice (Fig. 3F). These observations agree with the Western Blot data (Fig. 3C), wherein increased expression of angiogenesis-related proteins was noted.

## Discussion

BPD is a chronic lung disease commonly observed in preterm infants, characterised by vascularisation disorders and pulmonary vascular dysplasia. Preterm infants are highly susceptible to neonatal respiratory distress syndrome (NRDS) due to imperfect lung development, and hyperoxia therapy is an important means of treating NRDS. Due to the immaturity of antioxidant defence mechanisms, preterm infants are highly susceptible to oxidative stress in the lung, leading to oxidative damage (Kalikkot et al. 2017). In addition to pulmonary lesions, hyperoxia therapy is also an important cause of pulmonary hypertension and retinopathy in preterm infants, as well as lesions in other systemic systems (Green et al. 2023). In this study, we successfully established a mouse BPD model through hyperoxia exposure. The observed changes in lung histomorphology, such as pulmonary septal thickening, alveolar simplification, and variations in size, were consistent with classical BPD pathology, confirming the success of our model(Pozarska et al. 2017).

PVECs play a decisive role in pulmonary angiogenesis and contribute to the endothelial barrier function. The pulmonary vasculature actively promotes alveolar growth during lung development, which helps to maintain alveolar structure and promote lung development (Surate et al. 2017). However, hyperoxia exposure can lead to oxidative stress in PVECs, resulting in cellular oedema, dysfunction, and reduced survival and growth (Zhang et al. 2018). PECAM-1 is an abundant endothelial cell surface receptor that is highly enriched at endothelial cell junctions and mediates the proliferation and migration of PVECs. Therefore, we chose PECAM-1 to assess the status of PVECs and vascular development according to previous researchs(Hennigs et al. 2021; Liao et al. 2018; Lertkiatmongkol et al. 2016).. In this study, we observed decreased PECAM-1 protein level and reduced pulmonary vascular density in the lung tissues of BPD model mice exposed to hyperoxia. This suggests that hyperoxia inhibits PVEC proliferation, impacting pulmonary vascular development and vascularisation. VEGFA, a potent PVEC-specific mitogen and survival factor, promotes PVEC proliferation and differentiation, thereby facilitating pulmonary vasculature growth and remodelling. It serves as a key regulator of early pulmonary vascular development, contributing to neonatal hyperoxic lung injury (Wiszniak et al. 2021). Additionally, Ang-1, a vascular growth factor acting specifically on PVECs, cooperates with VEGFA to induce neonatal pulmonary vascularisation in vivo and maintain vascular stability (Kamp et al. 2022). In the present study, we found significant reductions in VEGFA and Ang-1 protein levels in the lung tissues of mice exposed to hyperoxia compared to those in the control group. This reduction in VEGFA and Ang-1 protein levels could hinder PVEC migration and tube formation, thus impeding pulmonary vasculature development under hyperoxia exposure conditions. Sudhadevi et al. found that a decrease in Ang-1 protein level was associated with the activity of PVECs and the impairment of alveolisation both in vitro and in mice (Sudhadevi et al. 2021). Therefore, together with our results, it is suggested that the decrease in VEGFA and Ang-1 protein levels, related to the proliferation of PVECs, represents impaired pulmonary vascular development under hyperoxia exposure.

Tregs are a group of T cells with immunosuppressive functions that play a crucial role in immune activation and developmental control of tissue damage in preterm infants (Pagel et al. 2020). Tregs have protective effects against PVECs in diseases such as pulmonary hypertension (Tamosiuniene et al. 2011). Our study revealed a decrease in Treg numbers in the lung tissues of mice exposed to hyperoxia, suggesting that hyperoxia inhibits Treg proliferation. Treg count was positively correlated with PECAM-1 protein level. The Treg and PECAM-1 expression are not close to 1. It may be related to the fact that multiple other factors are involved in BPD vascular endothelial injury together with Treg, such as endothelial progenitor cells, placental growth factor, and Ang-1/Tie-2 pathway. In previous studies, we found that M2 macrophages enrichment may have an important role in LPS induced BPD model mice by regulating type 2 immune responses (Mi et al. 2020). Research has found that Treg enhances autocrine signalling in macrophages by stimulating the production of IL-10 thereby causing inflammation to subside(Proto et al. 2018). However, it is unclear whether there is an interaction between Treg and macrophages in hyperoxia induced BPD, which requires further research. Moreover, Treg count was consistent with the trends observed in VEGFA and Ang-1 protein levels. This indicates that Tregs may protect the pulmonary vasculature by promoting PVEC proliferation. Thus, the impaired pulmonary vasculature development in BPD model mice may be linked to the attenuated protective effect of Tregs.

FOXP3, a nuclear transcription factor, is an important marker for Tregs (along with other classical markers such as CD4^+^, CD25^+^, and FOXP3^+^), which exert their immunosuppressive functions under its control (Wang et al. 2020). Kurebayashi et al. demonstrated that antagonism of FOXP3 expression in tumour vasculature decreases vascular density (Kurebayashi et al. 2021). Another study showed that in pulmonary hypertension, FOXP3 has also been associated with promoting pulmonary angiogenesis and development (Tian et al. 2021). Our study revealed consistent FOXP3 protein level and Treg proliferation in both control and hyperoxia-exposed C57 mice, suggesting that the protective effects of Tregs on the pulmonary vasculature are mediated through FOXP3.

IRF4, a member of the IRF family, primarily expressed in immune cells, serves as a co-transcription factor for Tregs. It plays roles in various immune functions, including cell development, differentiation, proliferation, and apoptosis, as well as the regulation of intrinsic and adaptive immune responses induced by pathogens (Nam et al. 2016). IRF4 is involved in the pathological process of a variety of inflammatory diseases, including the promotion of interleukin(IL)-6 production in inflammatory bowel disease, which induces STAT3 expression in T lymphocytes to promote apoptosis followed by intestinal inflammation (Zhu et al. 2016). Furthermore, IRF4 deficiency reduces inflammation and renal fibrosis following acute kidney injury induced by folic acid (Sasaki et al. 2021). Studies have also shown that IRF4 is involved in regulating immune responses such as oxidative stress in the lungs, and lung intrinsic lymphocytes depend on IRF4 to exert pro-inflammatory effects. For instance, *IRF4* knockdown in mice markedly attenuates pro-inflammatory responses in response to external environmental stimuli, suggesting the involvement of IRF4 in inflammatory responses in the lungs (Liu et al. 2018). In addition, IRF4 can be expressed in vascular smooth muscle cells, regulating vascular wall integrity and functions. In our hyperoxia-induced BPD model, we observed significantly increased IRF4 protein levels in lung tissues, especially after 14 vs. 7 days of hyperoxia exposure. This suggests that upregulation of IRF4 may be involved in BPD development.

**Fig. 4:**
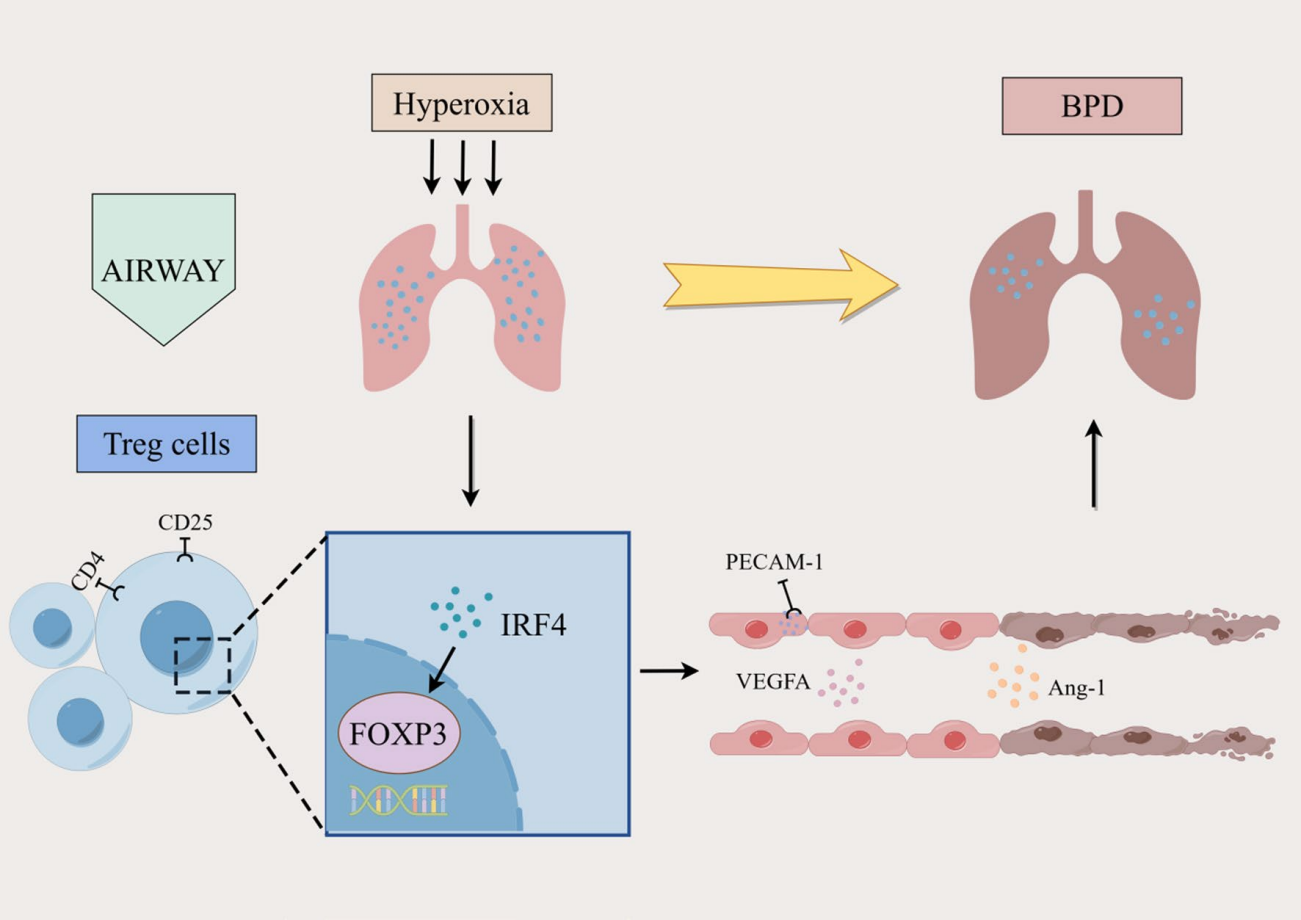
Mechanism diagram. Hyperoxia increases IRF4 levels and affects the protective effect of Tregs on the proliferation of PVECs and pulmonary vascular development by inhibiting FOXP3 levels, which ultimately leads to impaired pulmonary vascular development and BPD

FOXP3 and IRF4, as two co-transcription factors of Tregs, are jointly involved in the signalling pathway to regulate their expression. It has been observed that FOXP3 is significantly upregulated in *IRF4*^−/−^ Th cells cultured in the co-presence of IL-21 and transforming growth factor-β (Huber et al. 2008). Decreased FOXP3 expression and enhanced IRF4 expression were also detected in a mouse model of allergic asthma (Übel et al. 2014). On this basis, we also investigated the interaction between IRF4 and FOXP3 in the lung tissues of BPD model mice. We found a significant negative correlation between IRF4 and FOXP3 protein levels, suggesting that IRF4 exerts an inhibitory effect on FOXP3. This inhibition may influence the protective effect of Tregs on PVEC proliferation and pulmonary vascular development, contributing to BPD development (Fig. 4).

To explore this further, we used CRISPR/Cas9 technology to knock down the *IRF4* gene in mice and conducted hyperoxia exposure experiments. Knocking down *IRF4* resulted in increased FOXP3 protein level, promoting Treg proliferation. Additionally, *IRF4*^−/−^ mice exposed to hyperoxia exhibited improved lung histopathology, enhanced lung structure organisation, increased PECAM-1 protein level, higher lung vascular density, and elevated levels of angiogenesis-related proteins VEGFA and Ang-1. These results indicate that *IRF4* knockdown mitigates lung injury and impaired lung vascular development under hyperoxia conditions.

In this study, we focused on the protective effect of IRF4 on the pulmonary vasculature through the regulation of FOXP3 affecting Treg in BPD model mice. However, there may be some limitations in this experiment. We were unable to co-culture Treg with PVECs or deplete Treg at the cellular level to observe functional changes such as proliferation and migration of PVECs. In addition, the experimental approaches have been performed in lung, as an organ, and therefore exposed to multiple systemic signals, which control tissue response. In the future, using lung organoid model to study the cellular and molecular regulatory mechanisms in BPD, understanding the interactions among multiple lung cells, can better reveal the pathological and physiological processes of the disease.

## Conclusion

In summary, our findings suggest that reduced PVEC proliferation and impaired pulmonary vascular development in hyperoxia-induced BPD model mice may be associated with IRF4's inhibitory effect on FOXP3. Knocking down *IRF4* can regulate FOXP3 and Tregs, improving lung development and mitigating impaired lung vascular development. This offers a new strategy for potential clinical treatments of BPD.

## Data Availability

All data generated or analysed during this study are included in this published article and its supplementary information files.

## Abbreviations

BPD: Bronchopulmonary dysplasia
PVECs: Pulmonary vascular endothelial cells
PECAM-1: Platelet-endothelial cell adhesion molecule 1
VEGFA: Vascular endothelial growth factor A
Ang-1: Angiopoietin-1
Tregs: Regulatory T cells
FOXP3: Forkhead box protein P3
IRF4: Interferon regulatory factor 4
HE: Haematoxylin–eosin
IHC: Immunohistochemistry
BSA: Bovine serum albumin
SDS-PAGE: Sodium dodecyl sulfate–polyacrylamide gel electrophoresis
Co-IP: Co-immunoprecipitation
IF: Immunofluorescence
PBS: Phosphate-buffered saline
DAPI: 4′,6-Diamidino-2-phenylindole
CD4^+^: Cluster of differentiation 4-positive
CD25^+^: Cluster of differentiation 25-positive
CRISPR/Cas9: Clustered regularly interspaced short palindromicrepeats/CRISPR-associated protein 9
SD: Standard deviation
IL: Interleukin
NRDS: Neonatal respiratory distress syndrome
